# Genomic Selection in an Outcrossing Autotetraploid Fruit Crop: Lessons From Blueberry Breeding

**DOI:** 10.3389/fpls.2021.676326

**Published:** 2021-06-14

**Authors:** Luís Felipe V. Ferrão, Rodrigo R. Amadeu, Juliana Benevenuto, Ivone de Bem Oliveira, Patricio R. Munoz

**Affiliations:** ^1^Blueberry Breeding and Genomics Lab, Horticultural Sciences Department, University of Florida, Gainesville, FL, United States; ^2^Hortifrut North America, Inc., Estero, FL, United States

**Keywords:** genotyping by sequencing, sequencing depth, allele dosage, plant breeding, molecular marker, fruit quality, independent validation, genomic prediction

## Abstract

Blueberry (*Vaccinium corymbosum* and hybrids) is a specialty crop with expanding production and consumption worldwide. The blueberry breeding program at the University of Florida (UF) has greatly contributed to expanding production areas by developing low-chilling cultivars better adapted to subtropical and Mediterranean climates of the globe. The breeding program has historically focused on recurrent phenotypic selection. As an autopolyploid, outcrossing, perennial, long juvenile phase crop, blueberry breeding cycles are costly and time consuming, which results in low genetic gains per unit of time. Motivated by applying molecular markers for a more accurate selection in the early stages of breeding, we performed pioneering genomic selection studies and optimization for its implementation in the blueberry breeding program. We have also addressed some complexities of sequence-based genotyping and model parametrization for an autopolyploid crop, providing empirical contributions that can be extended to other polyploid species. We herein revisited some of our previous genomic selection studies and showed for the first time its application in an independent validation set. In this paper, our contribution is three-fold: (i) summarize previous results on the relevance of model parametrizations, such as diploid or polyploid methods, and inclusion of dominance effects; (ii) assess the importance of sequence depth of coverage and genotype dosage calling steps; (iii) demonstrate the real impact of genomic selection on leveraging breeding decisions by using an independent validation set. Altogether, we propose a strategy for using genomic selection in blueberry, with the potential to be applied to other polyploid species of a similar background.

## Introduction

Blueberry (*Vaccinium corymbosum* and hybrids) is recognized worldwide for its health benefits due to the high content and diversity of polyphenolic compounds (Kalt et al., 2020). Such health-related attributes have resulted in an increased demand for blueberries, as it has become a crop with one of the fastest growths in production trends, with an increase of 142% of its production in the last 10 years (FAOSTAT, 2021). In this sense, the blueberry breeding program at the University of Florida (UF) has had a major contribution to the expansion of production areas. Starting in the 1950s, the UF blueberry breeding program led to pioneering hybridizations between high-quality US northern adapted species (*Vaccinium corymbosu*m) and endemic US southern species (e.g., *Vaccinium darrowii*), selecting for low-chill requirements to break the dormancy of flower buds (Sharpe and Sherman, 1971; Lyrene, 2000). The resulting breeding material and cultivars, known as southern highbush blueberries, established a new industry in Florida and multiple warmer regions worldwide, allowing a year-round supply of fresh blueberries for the global market.

Historically, like many others, the UF program used recurrent phenotypic selection with visual assessment of plants to select both new parents for crossing and genotypes for commercial testing (Cellon et al., 2018). Despite the success of the industry and the release of many cultivars in recent decades, the use of conventional methods results in low genetic gains per unit of time. Moreover, the autopolyploid nature of the crop, long juvenile phase, multi-year evaluations, large experimental areas, and the high sensibility to inbreeding depression make phenotypic selection costly and time-consuming. Remarkably, it can take up to 12 years to release a new cultivar using conventional tools (Lyrene, 2005). As DNA sequencing costs continue to decrease, genomics-based markers present an opportunity to accelerate the breeding process by achieving more accurate selection during earlier breeding stages. Therefore, the UF blueberry breeding program has been leading innovative genomics studies and procedures to fill two primary gaps in the blueberry breeding literature: understanding the genetic architecture of complex traits via genome-wide association studies (GWAS) and quantitative trait loci (QTL) mapping; and, at the practical level, performing genomic prediction based on molecular markers, a methodology popularly referred to as genomic selection (GS).

GWAS and QTL mapping are both tools for providing a biological elucidation of the genetic architecture, in which molecular markers spanning the entire genome are statistically tested for associations with phenotypes (Pritchard et al., 2000). While QTL analyses are usually performed using structured populations, GWAS increases the mapping resolution by using populations with low levels of linkage disequilibrium considering a deep history of recombination events. In blueberry, we recently detected candidate genomic regions and markers associated with different fruit quality traits (Ferrão et al., 2018) and flavor-related volatiles (Ferrão et al., 2020) via GWAS investigations; and we built a high-density linkage map and detected QTL associated to berry firmness (Cappai et al., 2020a). In counterpart, GS aims to predict breeding values by using all genome-wide markers simultaneously (Meuwissen et al., 2001). The underlying rationale is that most QTL will be in linkage disequilibrium with some of the markers used whenever the marker density is high enough. Therefore, the estimated effect of all markers will lead to accurate predictions of the genetic merit for a complex trait. We have recently shown the potential of GS in blueberry breeding under distinct modeling scenarios (de Bem Oliveira et al., 2019, 2020; Amadeu et al., 2020a; Zingaretti et al., 2020).

The autopolyploid nature of blueberry (2*n* = 4X = 48) imposes additional challenges for analyzing and interpreting genetic data. Autopolyploids possess genomes with multiple sets of homologous chromosomes, resulting in non-preferential pairing and potential polysomic inheritance during meiosis. Given the presence of higher allele dosage (i.e., the number of copies of each allele at a particular locus), a higher number of genotypic classes are possible (Gallais, 2003; Garcia et al., 2013; Dufresne et al., 2014). Thus, the inclusion of allelic dosage information on GS models could imply a more accurate estimation of breeding values by considering the additive effect of multiple copies of the same allele and the potential inheritance of dominance effects. However, accurate allele dosage calling on polyploids depends on a higher depth of coverage, increasing genotyping costs when using sequence-based genotyping platforms (Gerard et al., 2018; Caruana et al., 2019). After performing foundational studies on the importance of polyploid models, the inclusion of non-additive effects, and sequencing depth on allele dosage parameterizations, the UF blueberry breeding program is now on track to overcome the barrier a simple promise to make GS a reality.

Motivated by the potential to use GS to reshape traditional blueberry breeding, we herein revisited some of our previous studies and described the current achievements in blueberry. Thus, our contributions in this paper are three-fold: (i) summarize previous results on the relevance of model parametrizations, such as diploid or polyploid methods, and inclusion of additive and non-additive gene actions for prediction; (ii) assess the importance of accurate dosage estimation for genomic prediction under low and high sequencing depth scenarios; (iii) demonstrate the realized impact of GS over breeding cycles by using an independent validation set. Altogether, we anticipate challenges and directions for future studies in blueberry that could be applied to other polyploid and fruit species of a similar breeding background.

## Materials and Methods

### Populations and Phenotypic Data

The southern highbush blueberry populations used in this study were generated as part of the breeding program at the University of Florida. Two phenotypic datasets, referred to as *calibration set* and *testing set*, were used for different purposes.

The *calibration set* comprises a large breeding population already described in previous studies (Ferrão et al., 2018; de Bem Oliveira et al., 2019). Briefly, it consists of 1,837 individuals originating from 117 biparental crosses using 146 distinct parents. The population corresponds to early stages in the breeding scheme, and it was planted in a high-density nursery at the “Plant Science Research and Education Unit” in Citra, Florida. All phenotypic evaluations were conducted on ripe fruits collected from the beginning of April to mid-May. Fruit firmness (g^*^mm^−1^ of compression force), size (mm), and weight (g) were evaluated over two seasons (2014 and 2015), while soluble solid (°Brix) was evaluated only in 2015. Given the large representative population, all genomic prediction models reported in this study were calibrated using this dataset. The empirical best linear unbiased estimates (eBLUEs) were estimated for each genotype based on a linear model. Genotype and year were considered fixed effects, as described by Amadeu et al. (2020a). Hereafter, the eBLUEs for each trait were considered as our response variable in the genomic prediction analyses.

The *testing set* was used for independent validation in genomic prediction analyses. It comprises 280 advanced selections not originally included in the *calibration set*. These genotypes represent materials in advanced stages in the breeding program planted over 2013–2017 under commercial conditions. These genotypes were evaluated over several years (2014–2020), some of them (16 common genotypes) in different locations throughout Florida. As these phenotypes were collected from plants in different physiological phases and multiple environments, we adjusted the phenotypes using a linear model, including separate fixed effects for the year, location, and plant age. The eBLUEs of each genotype per trait were used as the phenotypic value in subsequent genomic prediction analyses. All phenotypic analyses were carried out using the ASReml-R software (Butler et al., 2009). Additional details about the *calibration* and *testing* datasets are reported in Supplementary Figures 1, 2.

### Genotyping

The *calibration set* was genotyped using the “Capture-Seq” approach described in Benevenuto et al. (2019). The genotyping of the *testing set* was also performed using “Capture-Seq,” considering 10,000 biotinylated probes of 120-mer at RAPiD Genomics (Gainesville, FL, USA). Sequencing was carried out in the Illumina HiSeq2000 platform using 150 cycle paired-end runs. To ensure that the same group of single nucleotide polymorphisms (SNPs) will be called in both *calibration* and *testing* sets, we included the next-generation sequence data from both sets under the same SNP calling pipeline. First, raw reads were cleaned and trimmed. Then, the remaining reads were aligned using Mosaik v.2.2.3 (Lee et al., 2014) against the largest scaffolds of each of the 12 homoeologous groups of *Vaccinium corymbosum* cv. “Draper” genome assembly (Colle et al., 2019). SNPs were called with FreeBayes v.1.3.2 using the 10,000 probe positions as targets (Garrison and Marth, 2012). Loci were filtered out applying the following criteria: minimum mapping quality of 10; only biallelic locus; maximum missing data of 50%; minor allele frequency of 1%; and minimum and maximum mean sequence depth of 3,750 across individuals, respectively. A total of 63,552 SNPs were kept after these filtering steps. Sequencing read counts per allele per individual were extracted from the variant call file using vcftools v.0.1.16 (Danecek et al., 2011) and subsequently used to investigate some practical questions implementation of genomic prediction in polyploids.

We first investigated the importance of accurate genotype calling for genomic prediction by testing *ratio* and *dosage* under high and low sequencing depth scenarios. For this purpose, we used the *calibration* set only in a 10-fold cross-validation scheme. For the *ratio* method, each genotypic score was computed as the ratio between the alternative and total read depth, as described by Sverrisdóttir et al. (2017) and applied in de Bem Oliveira et al. (2019). For the *dosage* method, genotypic classes were assigned probabilistically using the updog R package v.2.1.0 considering the “norm model” and prior bias equals zero (Gerard et al., 2018; Gerard and Ferrão, 2020). Both genotyping methods (*ratio* and *dosage*) were compared under scenarios of high sequencing depth (random sampling for the mean number of 60 reads – 60×) and low sequencing depth (random sampling for the mean number of 6 reads – 6×). Specifically, we assumed the sequencing reads of each allele (alternative or reference) for a given marker come from a multinomial distribution, with probability equal to the number of the reads divided by the total number of reads across all the alleles, markers, and individuals (*N*). Then, we sampled *N*/10 reads from this multinomial distribution. We performed this sampling 10 times, and each sampling result was used in a different cross-validation fold. To avoid an eventual confounding between the number of markers and the predictive ability over the four scenarios, we kept the same number of SNPs (63,552) across all scenarios. Therefore, in total, four scenarios were tested: *ratio_60x, ratio_6x, dosage_60x*, and *dosage_6x*.

For the real validation and implementation of GS in the blueberry breeding program, we used the actual read counts to estimate the allele dosage in the *calibration* and *test* sets according to the “norm model” in the updog 2.1.0 R package (Gerard et al., 2018; Gerard and Ferrão, 2020). The posterior probability modes were used as our genotypic score. After estimating the posterior mean per genotype, we filtered out markers with a proportion of individuals genotyped incorrectly (“prop_miss” <10%) and markers with an estimated bias higher than 0.13 and smaller than 7.38. Missing genotypes were imputed by the mean of each locus. A total of 48,829 SNPs were kept and used in genomic prediction for independent validations.

### Statistical Analyses

Single-trait linear mixed models were used to predict breeding values using the best linear unbiased prediction (BLUP) and restricted maximum likelihood approach (REML) to estimate variance components, as following: ***y = μ + Zu + e***; where **y** is a vector of pre-corrected phenotypic records for a particular trait; **μ** is the overall mean; **Z** is an incidence matrix linking observations in the vector **y** to their respective breeding value in the vector **u**. Normality was assumed for the additive and residual effects, where $u\sim MVN\left(0,G\sigma_{u}^{2}\right)$ and the residual variance $e\sim MVN\left(0,I\sigma_{u}^{2}\right)$. For the residual, **I** is an identity matrix; while $\sigma_{u}^{2}$ and $\sigma_{e}^{2}$ are the genetic and residual variance components. The matrix **G** denotes the genomic relationship matrix computed using the ratio genotypic score or the tetraploid allele dosages with the different sequencing depths described above. The matrices were estimated in the AGHmatrix v.2.0.0 R package (Amadeu et al., 2016). For the *ratio* implementation, we used the “ratio” option in the software that computes the relationship as ***G*** = ***ZZ***′/*h*, where **Z** is the mean-centered matrix of the molecular marker information (ratio values); and *h* is a scale factor, where $h=\overset{m}{\underset{i=0}{\sum }}s_{i}^{2}$ and $s_{i}^{2}$ is the variance of the vector *z*_*i*_ centered marker *i* (for more details, see de Bem Oliveira et al., 2019). For the *dosage* implementation, we used the additive relationship matrix based on VanRaden (2008) as described by de Bem Oliveira et al. (2019). All genomic prediction analyses were carried out using the rrBLUP package (Endelman, 2011). For comparison, predictions were also carried out using pedigree BLUP. Using the same linear mixed model, we computed the numerator pedigree-based relationship considering autotetraploidy and no double reduction (Kerr et al., 2012), using the AGHmatrix v.2.0.0 R package (Amadeu et al., 2016).

Predictive performances were assessed for the *ratio* and *dosage* methods under high (60×) and low (6×) sequencing depth scenarios using only the *calibration set* in a 10-fold cross-validation scheme. To this end, the *calibration set* was randomly divided into 10 groups, where one group was used as a validation test, while the remaining nine groups were used as training. Models were trained in the validation test using the genomic best linear unbiased prediction (GBLUP) approach. For each fold, predictive abilities were estimated using Pearson's correlation between genomic estimated breeding values (GEBVs) and the corresponding eBLUEs. We also evaluated the correspondence between the top 20 groups of individuals ranked using *dosage_60x* and the other scenarios. A *post-hoc* Tukey test (alpha = 0.05) was used for intergroup comparisons between the top 20 ranked genotypes.

For the independent GS validation over the breeding cycles, we assessed the robustness of our predictive model over different scenarios: (i) *across-stages* scenario refers to 114 individuals from the *calibration* set that were clonally propagated in 2014 and planted in a commercial condition in a single location, becoming the *testing* set – prediction accuracy in this scenario can demonstrate the potential losses when models are trained at earlier stages (high density) and used at late stages of selection (commercial condition); (ii) *general* scenario stands for models trained in the *calibration* set and predictions carried out in the *testing* data, in which the target phenotypic values were pre-corrected for year, location, and age fixed effects; (iii) *stratified* scenario comprises models trained in the *calibration set* that were tested for predictions across four regions in Florida (North-FL, Central-FL, South-FL, and Citra-FL) – in contrast to the *general* predictions, in this scenario the target phenotypic values were pre-corrected only for the year effect per region. In all scenarios, predictive performances were assessed via Pearson's correlation.

**Figure 1:**
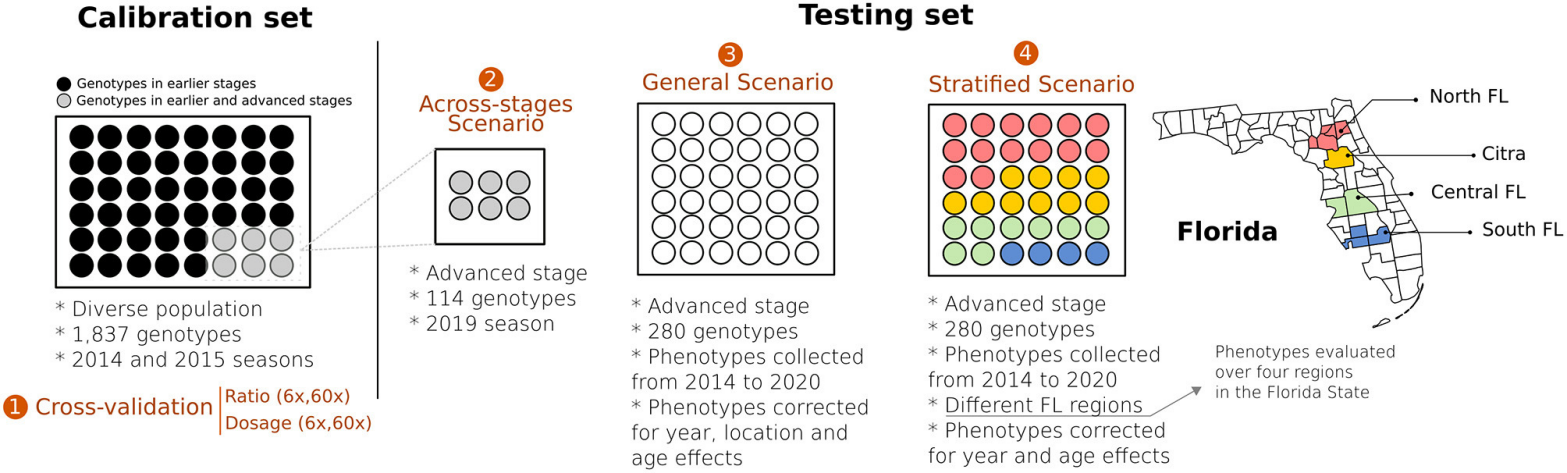
Schematic representation of four validation scenarios tested in blueberries. Calibration set represents a diverse group of genotypes representative of the UF blueberry breeding population. In Scenario 1, we used the calibration set in a 10-fold cross-validation scheme to test the relevance of genotyping calling (ratio vs. dosage) considering two different sequencing depths (6× and 60×). Scenario 2 (across-stages) represents a group of 114 individuals originally presented in the calibration set that were clonally propagated, moved to the advanced Stage of the breeding program, and phenotyped under commercial field conditions. Scenario 3 (general prediction) represents an independent group of 280 genotypes (testing set), evaluated under commercial conditions. The phenotypic values of the target individuals were pre-adjusted for the year, location, and age effects. Finally, in Scenario 4 (stratified prediction), we performed predictions over four regions of the State of Florida. To avoid potential model overfitting, we removed genotypes from the calibration set overlapped with the testing set.

A summary of all validation scenarios is illustrated in Figure 1. We complemented the predictive analysis for the stratified predictions by accessing the importance of genotype-by-environment interaction (GxE) via ANOVA. To this end, we considered 16 genotypes (checks) that were phenotyped over the four regions. We fitted a linear model considering the year, genotype, location, and the interaction between genotype and location (GxE) as fixed effects. ANOVA was performed in R (R Team, 2013) using the native *lm()* function.

## Results and Discussions

In the last two decades, GS has become a reality for many animal and plant breeding programs. Despite the optimism and proven efficacy, its wide implementation is still hindered by investment costs and the analytical skills required (Hickey et al., 2017). With that in mind, the UF blueberry breeding program initiated genomic studies on a large scale in 2013. First, we worked closely with genotyping companies to design customized genotyping platforms; we phenotyped and genotyped a large and multi-parental blueberry breeding population; we increased our computational resources; and finally, we adapted our breeding framework to incorporate genomics. During this process, the implementation of GS in a polyploid and outcrossing species proved challenging, particularly regarding the intrinsic biological complexities and the availability of genomic and computational tools (Mackay et al., 2019). In blueberry, for example, a high-quality genome assembly became available only in 2019 (Colle et al., 2019). As a result, about half of the capture-seq genotyping probes originally developed based on a draft genome assembly were discarded afterward based on the high-quality genome, without compromising genetic association and genomic prediction analyses (Benevenuto et al., 2019). We also explored additional optimizations to reduce costs regarding the number of individuals per family, the number of markers, and sequencing depth (de Bem Oliveira et al., 2020). Moreover, new genomics methods and tools have been developed in the last decade for the polyploid community, including allele dosage estimation, haplotype reconstruction, and the use of different relationship matrices (Bourke et al., 2018). Here, we presented the lessons we have learned so far for implementing GS in an autotetraploid and outcrossing species. We summarized previous results and also included novel findings relevant to the blueberry and polyploid community.

### Filling the Gaps: Phenotypic and Genotypic Selection in the Same Breeding Framework

**Figure 2:**
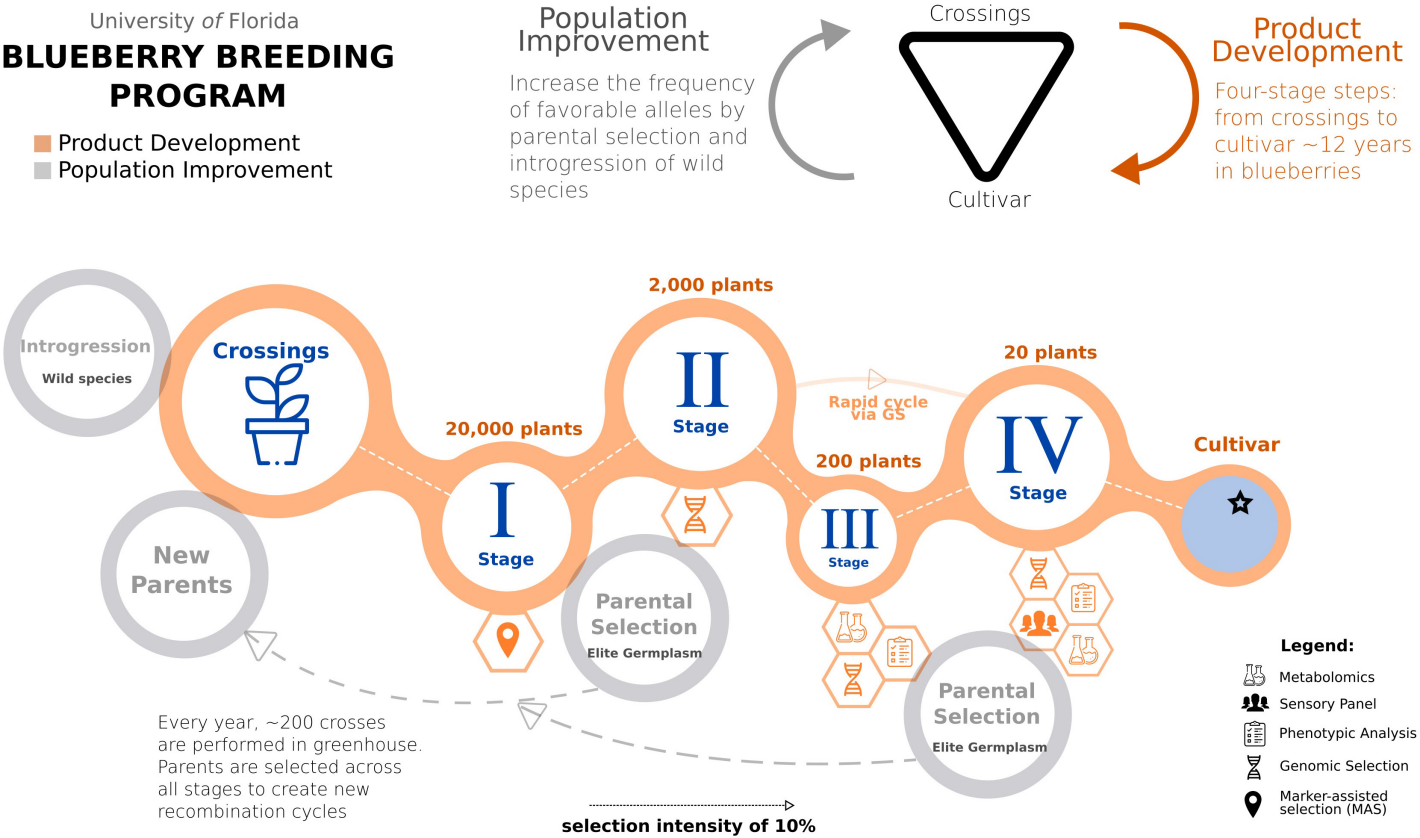
A schematic representation of the UF blueberry breeding program, integrating phenotypic selection and genomic prediction. The breeding process is conventionally organized in two integrated steps: population improvement and product development. A breeding cycle starts with crosses between outstanding parental genotypes. After that, several stages (I–IV) are required to evaluate the genotype performance. At Stage I, we will use marker-assisted selection targeting traits with simple genetic architecture. Genomic selection will be implemented in Stage II when GEBVs are computed. In advanced selections (Stages III and IV), high-quality phenotyping will be performed to leverage the calibration of genomic prediction models. At these stages, metabolomics and sensory panel analyses will also play an important role in flavor-assisted selections. In the end, elite materials are registered as clonally propagated cultivars. In addition, to shortening the time for product development, GS can be applied to move top-ranked plants directly from Stages II to IV, skipping at least 3 years of evaluation at Stage III. For population improvement, GS can assist in more accurate parental selection at early stages.

Blueberry is an outcrossing and clonally propagated crop, for which the breeding process can be conventionally organized in two central steps: population improvement and product development (Lyrene, 2005). First, population improvement is done to manage the frequency of beneficial alleles over time by selecting and crossing outstanding materials, as conceptualized in recurrent selection designs. Second, in parallel, product development consists of a series of trials in which potential candidates are evaluated over several years and locations, advancing across stages until selecting the best genotypes becomes a registered variety. In Figure 2, we illustrated these two key steps and how they are integrated into a four-stage selection design (from Stages I to IV) in the UF blueberry breeding program.

Annually, the blueberry breeding program performs more than 200 crosses, including parents selected among cultivars, elite material, and wild germplasm (Lyrene, 2005). From these crosses, about 20,000 seedlings are planted in non-replicated high-density nurseries (area of 0.2 ha), establishing the so-called Stage I. After 1 year, plants in Stage I are visually selected based on fruit size, color, scar, and using the breeder's “bite test” for flavor quality attributes. Approximately 10% of the original number of seedlings are kept after this first selection, and the unselected plants are removed from the field. To not exhaust genetic diversity, a minimal number of individuals per family are kept. However, given blueberry's long juvenile period, the availability of few berries, and the high competition in a high-density planting, it is difficult to phenotype for all traits and assess the individuals' full potential stage.

Additionally, the large number of individuals prevents genomic prediction at this stage, given the costs of genotyping. Therefore, at Stage I, we envision that marker-assisted selection (MAS) for traits with simple genetic architecture is a more feasible approach, and it is a current research line of the breeding program. In this regard, the example of MAS implementation in early selection stages is reported in strawberry (Gezan et al., 2017; Osorio et al., 2020).

After the first selection, ~2,000 genotypes pass to the second stage (Stage II). All plants stay in the same field plot, in high density. Further visual phenotypic evaluations are performed for the next 3 years. At this stage, we are implementing genomic prediction to increase genetic gains by improving phenotyping accuracy and selecting parents at early stages. Therefore, at Stage II, all plants will be genotyped. The GEBVs will be predicted for five fruit quality traits (soluble solids, titratable acidity, weight, size, and firmness), yield, and consumers panel liking scores. Using a selection index according to trait importance (Williams, 1962), we will perform GS to complement standard phenotypic descriptors and rank all genotypes. Different selection indexes are defined every year, depending on the traits and crosses performed, with yield and flavor traits usually receiving the highest weights. As routinely done, 10% of the 2,000 plants will be moved to the next stage (Stage III), where selected plants are clonally propagated and evaluated in a 15-plant clonal plot in a commercial field.

At Stage III, around 200 plants are more accurately phenotyped for more traits, using more fruits, clonal repetitions, and multiple years of evaluations in commercial conditions. Technically, all information collected at this stage will be used to feed the genomic prediction models. The UF blueberry breeding program has included new traits for routine phenotyping to meet the current demand from different marketable demands in recent years. For example, the use of volatiles for flavor-assisted selection has shown the ability to predict sensory perceptions by explaining 55% of the variation in overall liking scores (Colantonio et al., 2020). Given the high costs to perform sensory panels, we are incorporating metabolomics in the breeding pipeline to predict flavor ratings for many genotypes at Stage III (Gilbert et al., 2015; Colantonio et al., 2020; Ferrão et al., 2020).

In the last stage (Stage IV), around 15–20 plants selected from Stage IIIs with consistent and outstanding performances are propagated and planted at commercial trial sites across Florida. The different locations comprise two production systems according to the accumulation of chilling hours: evergreen and deciduous (Fang et al., 2020). To ensure accurate selection, phenotypic data is collected weekly and used to feed our genomic prediction models. Fruits from selected genotypes are also submitted to sensory panels, where blueberry consumers score flavor preferences. Elite selections from this final Stage are ultimately named, patented, and released as clonally propagated cultivars.

Altogether, the conventional breeding pipeline takes up to 12 years to evaluate the genotype merit of an individual to be released as a cultivar. With the implementation of genomic selection at the scope of the breeding program, the selection criteria can be more accurate than the visual phenotypic selection at Stage II. Moreover, it will shorten the time to select genotypes to become a parent in the next breeding cycle and advance to Stage III. In a typical recurrent selection breeding scheme, the parental selection is crucial (Lyrene, 2005). We have optimized this selection by ranking the GEBVs over the breeding cycles and seeking crosses that minimize inbreeding. Among the different tools available for mate allocation, we have recently implemented the algorithm described in the AlphaMate software with default parameters (Gorjanc and Hickey, 2018).

### “*Simplicity Is the Ultimate Sophistication*”^1^: On the Relevance of Additive GBLUP Models

When confronting the problem of modeling the relationship between molecular markers and variation in the observed traits, an important question to keep in mind is what statistical method could better describe this relationship (Ferrão et al., 2019). In recent years, we have investigated statistical and biological aspects underlying the implementation of genomic prediction in autopolyploid species, including (i) the importance of accounting for allele dosage in whole-genome statistical models (de Bem Oliveira et al., 2019); (ii) the relevance of multiple gene actions, including additive and non-additive genetic sources (Amadeu et al., 2020a; Zingaretti et al., 2020); and finally, (iii) the impact of sequencing depth of coverage, when sequence-based genotyping approaches are used (de Bem Oliveira et al., 2020).

Among the factors that differentiate diploid and polyploid analyses, resolving the allelic dosage of individual loci is one the most important. While in diploid organisms, only three genotypic classes are possible for biallelic markers, autotetraploids, like blueberry, can have up to five genotypic classes. Therefore, in theory, it is expected that statistical models accounting for the dosage effect could be more informative and provide a more realistic representation of the genetic complexity of a quantitative trait (Garcia et al., 2013). We first tested this hypothesis by contrasting polyploid and diploid parametrizations in GWAS studies (Ferrão et al., 2018), whereby a larger number of associations were observed under polyploid models. In a subsequent study, we investigated a similar assumption for genomic prediction (de Bem Oliveira et al., 2019). We tested GBLUP models using relationship matrices built in a tetraploid (Slater et al., 2016) and diploid (VanRaden, 2008) fashion.

Interestingly, both parametrizations resulted in similar performances for all traits tested. Furthermore, the similar predictive ability for diploid and polyploid parametrizations was also reported in other autotetraploid species (Lara et al., 2019; Matias et al., 2019), which ultimately reinforced the robustness of the predictive accuracy of GBLUP regardless of the ploidy parametrization used. These results are explained by the similarity between the genomic relationship matrices computed using diploid and autotetraploid parametrizations. Recently, we presented empirical evidence on this topic by showing that the estimation of molecular pairwise relatedness in both scenarios are highly correlated, in particular, under low-to-middle rates of heterozygosity (Amadeu et al., 2020b).

Besides the potential additive impact of allele dosages, dominance effects can also be heritable in polyploids and could improve the prediction of genetic values. Therefore, it is also reasonable to speculate that a greater number of alleles per locus may increase the range of genetic models to describe one-locus genotypic value by accounting for multiple dominance levels (Gallais, 2003). This is exemplified by the different models addressing the dominance effect proposed in the polyploid literature, including the use of digenic interactions (Endelman et al., 2018), the use of a general effect by assuming that each genotype has its effect (Rosyara et al., 2016; Slater et al., 2016), and the use of heterozygous parametrization (Enciso-Rodriguez et al., 2018). In blueberries, we tested the importance of such different gene actions in predictive studies. Although we have observed an improvement in the statistical goodness of fit when dominance effects are counted, this increment is not directly translated into predictive ability (Amadeu et al., 2020a). Hence, the additive model resulted in performance similar to models accounting for dominance effects, as it has been described for diploid species (Muñoz et al., 2014).

Given the genetic complexity of polyploids and the potentially higher intra- and inter-locus interactions, we also hypothesized that predictions could be improved by using deep learning techniques (Zingaretti et al., 2020). Through deep learning, we could take advantage of non-linearity assumptions to model the whole genetic merit of an individual. We used allo-octoploid strawberry and autotetraploid blueberry as our biological models and compared linear models and deep learning techniques for prediction to test this. We did not observe improvements of deep learning over traditional linear models for traits with presumably different genetic architectures in both species. The only exception was observed in a simulated data set. Deep learning performed better for traits with large epistatic effects and low narrow-sense heritability, which reinforced the high predictive ability of mixed models as prediction machinery.

Our last contribution to the practical implementation of genomic prediction in polyploids is the relevance of sequencing depth of coverage for genotyping methods based on next-generation sequencing. Sequencing depth refers to the number of reads sequenced at a given site in the genome. Low coverage datasets increase the chances of not sampling all homologous chromosomes at a given site for a given individual during sequencing. Thus, it could result in high rates of missing data, miscalled genotypes, and uncertainty of allele copy number in heterozygous genotypes (Clark et al., 2019). Some studies in polyploid crops have recommended increasing the sequencing depth to circumvent this issue, which implies higher costs of genotyping. For example, Bastien et al. (2018) and Uitdewilligen et al. (2013) suggested sequencing depths of 50X−80X for an accurate assessment of allele dosage in autotetraploid potatoes. In a recent study, we demonstrated that such numbers are quite conservative for genomic prediction. By combining a simple genetic parametrization (*ratio*) and low-to-mid sequencing depth (*6x*–*12x*), we achieved similar predictive accuracies as the ones obtained using higher depths for blueberry traits with different genetic architectures (de Bem Oliveira et al., 2020). In practical terms, reducing the amount of sequencing data will also reduce the costs of implementing GS or potentially genotyping more individuals under a fixed budget.

**Figure 3:**
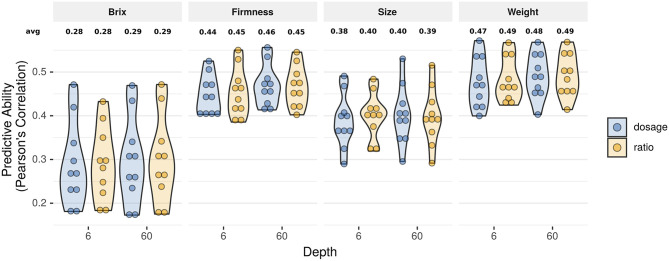
Violin plot with predictive ability considering two genotype calling approaches (dosage and ratio) under two sequencing depth scenarios (6× and 60×) for four fruit quality traits in blueberry using 10-fold cross validation. Each circle represents one cross validation fold result.

Despite the considerable advancements previously explored, the relevance of using more sophisticated algorithms for genotype calling and its impact on genomic prediction remains unexplored. Recently, several new methods have been developed to assign accurate allelic dosage of individual loci in polyploids (Garcia et al., 2013; Gerard et al., 2018; Pereira et al., 2018; Clark et al., 2019). In this paper, we compared predictive abilities. We confirmed that low-to-mid sequencing depth and ratio parametrization could be used to rank GEBVs with similar predictive performance (Figure 3 and Supplementary Table 2) and genotypic ranking (Table 1). Nonetheless, despite the attractive simplicity of using the ratio and low-sequencing depth, such results are only valid for prediction analysis (de Bem Oliveira et al., 2019, 2020). Importantly, there is no empirical evidence that setting the parameters to these levels could work for inferential studies such as GWAS, population genomics, linkage, and QTL mapping. In this sense, an important counterpoint was recently reported in hexaploid sweet potato. Higher sequencing depths and accurate dosage calling improved the ultra-dense linkage map and posterior QTL analysis (Gemenet et al., 2020; Mollinari et al., 2020). For GWAS, we observed large rates of false-positive associations when analyses were performed using low sequencing depth associated with the ratio parametrization (results not shown). Herein, we systematically observed large biases when relationship matrices were constructed using the *ratio_6x* approach (Supplementary Figure 4 and Supplementary Table 4).

**Table 1:** The number of genotypes matching the top 20 rankings using the dosage_60× method as the benchmark, under 10-fold cross-validation.

**Method**	**Depth**	**Firmness**	**Size**	**Weight**	**Brix**
Dosage	6×	16.5^b^	16.9^b^	16.3^b^	16.4^b^
Ratio	6×	16.2^b^	15.6^c^	16.2^b^	15.3^b^
Ratio	60×	18.8^a^	18.3^a^	18.6^a^	18.7^a^

*A post-hoc Tukey test (alpha = 0.05) was used for intergroup comparisons over the scenarios. Cells with the same letter represent non-statistically different groups for the given trait (column)*.

Our results suggest that the use of traditional GBLUP is robust enough for genomic prediction, even under simplistic assumptions. This fact has long been discussed in the specialized literature and has raised questions on the contribution of linkage disequilibrium between QTL and markers vs. the relationship information to GS (Habier et al., 2013).

### How Does Genomic Prediction Work in a Real Validation Population?

**Figure 4:**
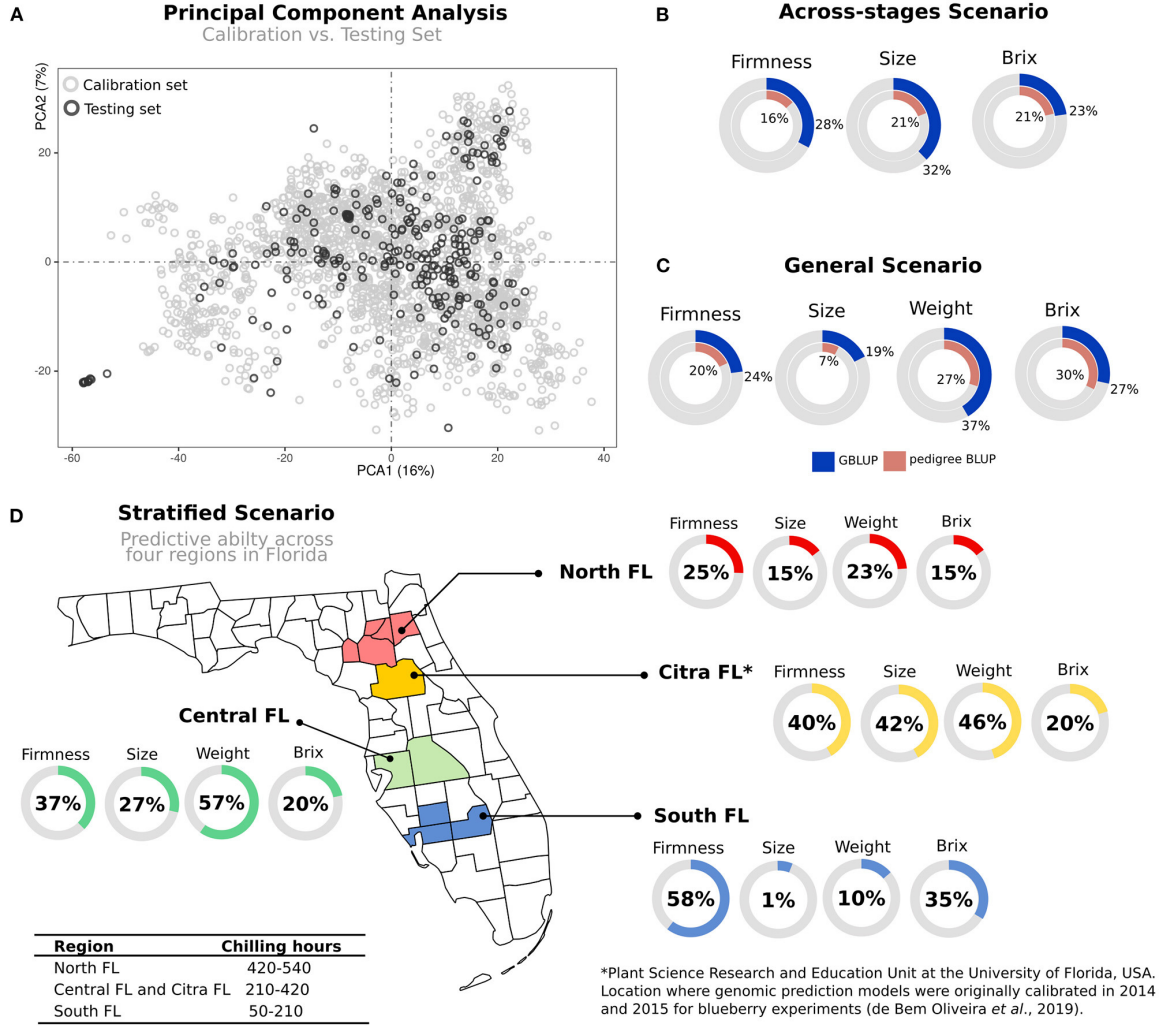
Genomic prediction on independent validation scenarios. **(A)** Principal component analysis (PCA) of two blueberry populations: *calibration* set represents the trained set, where genomic prediction models were originally calibrated; and *testing* set comprising additional 280 individuals used for testing. **(B)** Across-stages prediction: predictive ability was measured in a subset of 114 individuals included in the calibration set, but phenotypes were collected in advanced selection stages. **(C)** General prediction: the predictive ability of the testing set after training the models in the calibration set. **(D)** Stratified predictions: after training the models in the calibration set, individuals in the testing set were predicted using phenotypes collected over four macro-regions in the Florida State, which are under different chilling hour accumulation. All predictive abilities were expressed as percentage values.

While we have investigated several statistical and computational aspects related to GS in blueberry, it is still unknown how accurate the predictions will be across breeding cycles, with plants in different phenological stages and locations. This scenario came to be called “true validation” and involves the use of independent populations. We investigate it by dividing our prediction analyses as following: models calibrated in 2014 and 2015 using plants in Stage II were used for genomic predictions of individuals at Stages III and IV. Both data sets share genetic similarity (Figure 4A).

For independent validations, we tested different scenarios in which GS could be applied (Figure 1). First, we focused on validations across breeding stages. To this end, we used the *calibration* test—originally evaluated in Stages II—to predict a subset of individuals that were cloned and planted in an advanced stage (Stages III). When compared to within-sample cross-validation schemes, as originally reported by de Bem Oliveira et al. (2019) and Amadeu et al. (2020a), lower predictive accuracies were observed (Figure 4B). These results mainly highlight (i) the importance of collecting better phenotypic data and (ii) the influence of plant management. Remarkably, most of the phenotypic traits measured in the *calibration* set were collected from five berries per genotype, while on Stage III, we used 25 berries per genotype. Furthermore, genotypes in Stage II are planted in high-density nurseries with phenotypes collected in plants that are still in their juvenile phase. At the same time, Stages III are grown under commercial conditions and evaluated over several years.

A second predictive scenario tested the relevance of *calibration* tests at early stages to predict independent genotypes in advanced stages that were more extensively phenotyped. The results for most fruit-quality traits confirm the importance of genomic information (*general* predictions) over pedigree-based methods (Figure 4C). However, compared with predictions using within-sample cross-validation schemes, we also observed a reduction in the predictive results (Supplementary Table 3) (de Bem Oliveira et al., 2019; Amadeu et al., 2020a). This decline in predictive performance in true validation is expected due to differences in the allele frequencies over populations, variation in linkage disequilibrium patterns, and GxE interactions (Habier et al., 2013).

In the third scenario, a more challenging exercise was to measure how predictive ability varies across regions in the State of Florida (s*tratified* predictions, Figure 4D). Higher predictability was observed for Citra and Central-FL, the closest regions where the models were originally trained. In counterpart, plants evaluated in the South-FL showed, on average, lower predictability performances. Despite the small number of genotypes included in this analysis, these results provide insights into the importance of GxE interaction for GS in blueberry. We further explored this hypothesis by using a group of 16 common genotypes (checks) evaluated over the four regions. The results confirmed the significance of the GxE effect for most of the traits (Table 2 and Supplementary Figure 3), with the plants evaluated in South-FL showing the most contrasting values. It is noteworthy that blueberry locations in South-FL are grown under an evergreen production system, under less chilling hours, and are focused on preventing defoliation during the winter months (Fang et al., 2020). On the other hand, Citra, Central-FL, and North-FL regions are grown under the deciduous production system, where leaves are dropped during the winter. Such differences in the production systems could drive the largest disparity observed at South-FL compared with the other regions.

**Table 2:** Mean and standard deviation (in parenthesis) of four fruit quality traits were evaluated in advanced stages of the blueberry breeding program at four Florida regions.

**Location**	Firmness (g * mm^−1^)	**Size (mm)**	**Weight (g)**	**° Brix**
North FL	248 (32.8)	18.0 (1.79)	2.57 (0.641)	11.3 (1.31)
Citra	245 (42.0)	17.0 (2.22)	2.34 (0.691)	10.9 (1.33)
Central FL	244 (29.3)	17.7 (1.46)	2.29 (0.491)	11.8 (1.27)
South FL	251 (33.4)	17.4 (1.34)	2.21 (0.549)	12.0 (1.91)
GxE (*p*-value)^*^	0.007	0.0002	0.005	0.47

* *p-values associated to genotype-by-environment interaction (GxE) were computed using a linear model and ANOVA, where season, genotype, location, and the interaction between genotype and location (GxE) were fitted as fixed effects*.

*Values were computed using 16 common genotypes (checks)*.

The results from independent validations allow us to draw some practical conclusions. First, even with low-to-moderate predictive accuracies, GS is still encouraging. For example, soluble solids and firmness are both traits treasured by consumers, for which routine phenotyping is expensive and time-consuming for large populations, like Stage IIs. Ranking plants based on their GEBVs proved to be a better alternative than any other criteria historically used throughout UF blueberry breeding program (pedigree or visual selection). More accurate phenotypic data to annually recalibrate the model also has the potential to improve predictability.

### Unifying Biological Discoveries and Predictions

Genomic information can also provide new opportunities to integrate biotechnology and quantitative genetics into modern breeding programs, creating platforms for both deliveries of new products and biological discovery (Hickey et al., 2017). For example, in blueberry, biological discoveries have been addressed via QTL mapping (Cappai et al., 2020a) and GWAS studies (Ferrão et al., 2018, 2020) for multiple fruit quality traits. Unifying such discoveries with prediction is challenging, but it has been addressed under three different avenues: (i) use of GWAS discovered QTL as fixed effects on GS models; (ii) incorporating markers (or QTL) in MAS designs, and (iii) using genome-editing technology to speed up breeding.

In a strategy called “GS *de novo* GWAS,” we explored the importance and applicability of GWAS findings for prediction using the significant GWAS hits as fixed effects in GS models, considering independent datasets. For oligogenic traits, like some flavor-related volatiles, we achieved an increase of more than 20% in predictive ability compared with traditional GS methods (Ferrão et al., 2020). Using a similar strategy, gains in predictive performance have also been reported in other crops, such as maize (Bernardo, 2014; Rice and Lipka, 2019), wheat (Sehgal et al., 2020), and rice (Spindel et al., 2016). Alternatively, we have investigated further modeling strategies to accommodate biological information into the predictive models. For example, the use of Bayesian strategies that could accommodate SNPs with larger effect by using different prior distributions (Erbe et al., 2012; Gianola, 2013; Zhou et al., 2013); and GBLUP models that could weight variants previously selected either via association analysis or using bioinformatic pipelines (Su et al., 2014; Zhang et al., 2016; Liu et al., 2020; Ren et al., 2021).

Another potential strategy is to use target markers associated with important traits for MAS during Stage I of the blueberry breeding program. Such a strategy could be used for the early selection of plants still in the seedling stage. Acknowledged by their simple genetic architecture, we showed that few markers could yield reasonable predictive accuracies of volatile emission and, thus, leverage flavor selection (Ferrão et al., 2020). We envision that MAS can also be implemented for other oligogenic traits. In this regard, we have been conducting other GWAS and QTL mapping studies for disease resistance, such as anthracnose (*Colletotrichum gloeosporioides*) and bacterial wilt (*Ralstonia solanacearum*). A similar strategy has been implemented in strawberries (Gezan et al., 2017; Osorio et al., 2020) and other fruits (Iezzoni et al., 2020). However, for MAS to be applicable for thousands of plants, cheap and fast DNA extraction and targeted SNP genotyping assays should be optimized. We are currently testing high-resolution melting (HRM) and competitive allele-specific PCR (KASP) assays to validate and implement MAS for volatiles.

Gene editing is another attractive technology with the potential to have significant effects on the breeding program. Aside from the use of CRISPR-Cas9 for validating candidate genes identified via GWAS or QTL studies, some simulations have recently shown that genome editing can double the rate of genomic gain when coupled with genomic prediction, compared with GS conducted in isolation (Noman et al., 2016; Hickey et al., 2017). However, to our knowledge, there is only one study of CRISPR-Cas9 targeted mutagenesis in blueberry (Omori et al., 2021). At the UF blueberry breeding program, we have advanced our understanding of the best tissue culture practices and most effective transformation markers (Cappai et al., 2020b), laying the ground for CRISPR/Cas9 genome editing implementation in our breeding program. Using this technique, we can also take advantage of the knowledge accumulated from model crops to introduce novel allelic diversity in orthologs and accelerate the domestication process.

## Conclusions

The implementation of GS has already changed the UF blueberry breeding program routine by reorganizing how we collect genotypic and phenotypic information and analyze data to rank the material to advance stages and breed in the next cycles. Our previous studies on GS were fundamental to define the most cost- and time-effective methods for model parameterization and genotyping. The main lessons learned can be conveniently divided into different areas. Statistically, despite the numerous algorithms for prediction—many of them more elegant at the biological and computational level—the use of additive effects under a linear mixed model framework (GBLUP) showed the best balance between efficiency and accuracy. Considering the particularities of autopolyploid genetic data, we showed that for GS, low depth of sequencing (6×-12×) simplifies the allele dosage information (i.e., diploidization and ratio) resulted in similar prediction accuracies as those obtained using more refined scenarios. Finally, the genomic prediction was incorporated in a recurrent selection breeding scheme at the practical level, whereby variety development and populational improvement run in parallel. So far, GEBVs have been primarily used for parental selection to increase genetic gains while keeping the genetic diversity. A more objective reduction in the number of years to develop a cultivar would be selecting the top-ranked genotypes from Stage II directly to IV, skipping at least 3 years of evaluations at Stage III.

## Future Directions

Finally, we highlight some challenges and opportunities for further studies in blueberries. First, recalibrating the model with more accurate phenotypic data can yield better predictive ability. In this sense, phenomics is also a cutting-edge area of research that could leverage the number of traits and samples collected during a season and improve the quality of phenotypic data. For example, yield is a complex and time-consuming trait to be phenotyped over the season. We envision that image-based phenotyping may aid in evaluating yield and other traits, such as plant architecture and diseases. For the future, it would also be important to incorporate additional statistical checks (common genotypes) across years and locations to understand better the effects of GxE interaction on genomic predictions and recalibrate our models according to the environmental targets. On integrating multi-omics data, we expect that we will predict flavor preferences through volatile quantification and perform an early selection for more flavorful cultivars. Statistically, testing new algorithms for mate allocation and using haplotypes for prediction and imputation methods are some potential areas that could further improve genomic predictions.

## Data Availability Statement

The original contributions presented in the study are included in the article/Supplementary Material, further inquiries can be directed to the corresponding author.

## Author Contributions

PM and LF conceived and supervised the study. JB coordinated the collection and genotyping of the samples. IB coordinated the data collection for real validation. LF and RA analyzed and interpreted the phenotypic and genomic selection results. LF wrote the paper and included the revision from all authors. All authors read and approved the final version of the manuscript for publication.

## Conflict of Interest

IB was employed by company Hortifrut North America, Inc.; however, she participated in this research while at University of Florida. The remaining authors declare that the research was conducted in the absence of any commercial or financial relationships that could be construed as a potential conflict of interest.

## Footnotes

^1^Quote by Leonardo da Vinci.

## Abbreviations

UF: University of Florida
GEBV: genomic estimated breeding value
GWAS: genome-wide association study
QTL: quantitative trait loci
eBLUE: empirical best linear unbiased estimate
SNP: single nucleotide polymorphism
GxE: genotype by environment interaction
MAS: marker-assisted selection
GS: genomic selection
GBLUP: genomic best linear unbiased prediction.
