# Supplementary material for: Genomic Selection in an Outcrossing Autotetraploid Fruit Crop: Lessons From Blueberry Breeding
Source: Front Plant Sci. 2021 Jun 14;12:676326. doi: 10.3389/fpls.2021.676326 (PMC8236943; doi:10.3389/fpls.2021.676326)
Supplement: Supplementary file 1 [file Data_Sheet_1.docx]

Supplementary Material

**1) CALIBRATION SET**

**1.1) Plant Material and Phenotypic Data**

The populations used in this study were generated as part of the Blueberry Breeding Program at the University of Florida. Two populations were reported and named as *calibration* and *testing* sets.

The *calibration* set was is composed of 1,837 individuals derived from 117 controlled crosses using 146 distinct parents made in February 2011. All crosses were performed following the typical phenotypic recurrent selection pipeline, in which outstanding genotypes are annually selected from a broader germplasm pool and used as parents in new breeding cycles. Inbreeding is minimized by checking pedigree records. Moreover, no individual parent was used more than three times.

Seeds were extracted from the resulting mature fruit, cold-stratified for 5 months, and planted as a family bulk in 2 L pots in a greenhouse in November 2011. In the sequence, to compose the so-called Stages I, one hundred seedlings from each family were transplanted to larger pots in January 2012 and planted in May 2012 in a high-density nursery (~20,000 plants per 0.2 ha) at the Plant Science Research and Education Unit (PSREU) in Citra, Florida. In May 2013, after visual selection, undesirable genotypes were removed from the high-density nursery, leaving 5–32 plants from each family to advance in the breeding program. The remained population (so-called Stages II) was originally composed for 1996 individuals that were tagged and labeled to insure proper identification. Genotypes that did not follow the quality-control specifications for genotyping where excluded. Therefore, a total of number of 1,837 genotypes were considered in the calibration test.

Phenotypic data for the *calibration* set were collected in 2014 and 2015, when plants were 2,5 and 3,5 years of age, respectively. Fruit traits were determined using five randomly berries from each genotype, with few exceptions. Berries were harvested when the fruit were fully mature and only berries that exhibited picking quality, and had no visual, pathogen, insect, or bird damage were chosen. All these details about the breeding population, experimental design and phenotypic analyses were originally presented by Cellon et al. (2018).

**1.2) Population Structure**

Population structure and genetic parameters of the calibration set were originally described by Ferrão et al. (2018). Briefly, using molecular markers we observed a heterozygosity estimated of 0.42. As an outcrossing species with early-acting inbreeding depression, higher levels of heterozygosity are expected for the species. The inference of population structure was performed via Principal Component Analysis (PCA) and Discriminant Analysis of Principal Components (DAPC) using the tetraploid marker-based relationship matrix. The inspection of both PCA and DAPC results are indicating no evidence of population structure that would justify the use of correction approaches or an eventual stratification of the original population for predictive studies. Finally, at the significance threshold (r2 = 0.2), the LD decay presented significant correlation between markers 80 Kb apart. For more details, please see Ferrão et al. (2018) where these results were visually presented and discussed in the context of plant breeding.

**2) TESTING SET**

**2.1) Plant Material and Phenotypic Dispersion**

The *testing* set is a more complex dataset including plants from different ages (Stage III planted in 2014 and Stages IVs planted 2013-2017), measured over 2014-2020, some of them (16 genotypes) in multiple locations (four macroregions in Florida). The *testing* set is composed of 280 individuals planted and evaluated under commercial conditions. The advanced selected genotypes were clonally propagated and evaluated in a 15-plant clonal plot. Commercial plant spacing include 2.5 by 9 feet, resulting in 1,936 plants per acre. Two group of phenotypes were collected depending on the prediction scenario tested.

For the so-called *across-stages* prediction, a total of 114 genotypes originally evaluated in the calibration set were selected in 2013, cloned and installed in commercial conditions at the North FL region in 2014 (Stage III). In 2019, when the plants were 4 years of age, they were evaluated for fruit quality-traits. Fruit traits were determined using 25 randomly berries from each genotype. Berries were harvested when fruits were fully mature, with no visual, pathogen, insect, or bird damage. Figure S1 contrasts the phenotypic distribution of the same 114 genotypes for multiple fruit quality traits when they were evaluated in high-density nurseries (Stage II) in 2014 and 2015 (as part of the *calibration* set); and when they were in Stage III where they were cloned, installed in commercial conditions, and evaluated in 2019 (as part of the *testing* set).

**
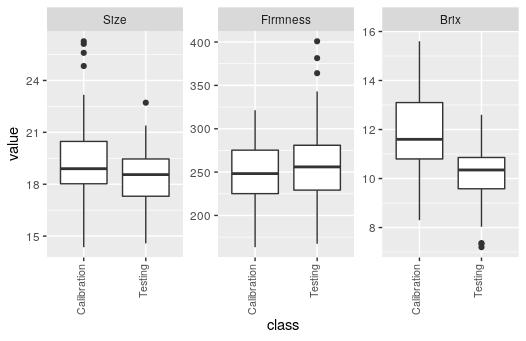
Figure S1**. Phenotypic variation observed for three fruity-quality traits among 114 common genotypes evaluated in the *calibration* and *testing* datasets. Firmness (g mm -1) and fruit diameter (mm) were measured simultaneously using a FirmTech II equipment. The soluble solids content (°Brix) was measured using a digital pocket refractometer (Atago U.S.A, Inc., Bellevue, WA) from 300 mL blueberry juice.

In the *general* scenario, the total testing set (280 advanced selections) was used. Those genotypes were evaluated from 2014 to 2020 in four main regions in the Florida State: North FL, Citra FL, Central FL, and South FL. The number of data points collected over the years across the four main regions is represented in the Table S1. As might be observed, Citra and North-FL are the main experimental locations, where most genotypes were phenotyped over the last 5 years. Since 2018, the Blueberry Breeding Program has increased the number of evaluations in the evergreen production system and therefore including new field trials in regions at the South of the Florida State.

Table S1. Number of data points collected over six years by the Blueberry Breeding Program in four main regions of the Florida State for 280 different genotypes.

| **Region** | **2014** | **2015** | **2016** | **2018** | **2019** | **2020** |
| --- | --- | --- | --- | --- | --- | --- |
| North FL | 0 | 0 | 5 | 26 | 274 | 39 |
| Citra | 6 | 6 | 3 | 15 | 94 | 45 |
| Central FL | 0 | 0 | 0 | 13 | 21 | 13 |
| South FL | 0 | 0 | 0 | 10 | 15 | 0 |

The phenotypic data distribution for soluble solids, firmness, size and weight across the four locations are shown in Figure S2. For all fruit quality traits, the empirical distributions were reasonably symmetric. On average, genotypes evaluated in the North-FL showed the largest values of firmness, size, and weight. In contrast, the highest values for soluble solids were observed in South-FL. It is noteworthy that not all environments have the same genotypes, and the data is highly unbalanced.


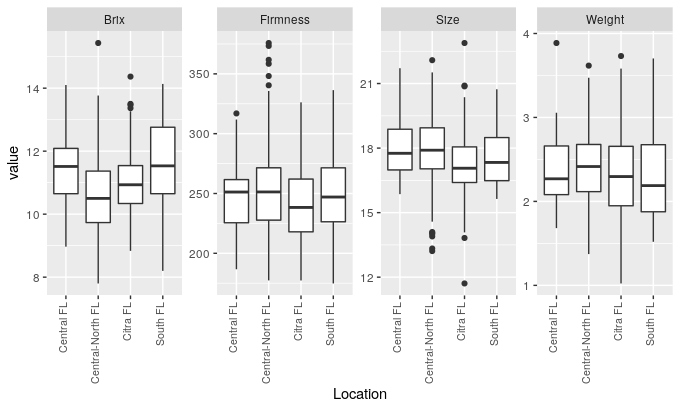


Figure S2. Phenotypic variation observed for four fruity-quality traits across genotypes evaluated over four regions at the Florida State: Central FL, Citra, North FL and South FL. Firmness (g mm -1) and fruit diameter (mm) were measured simultaneously using a FirmTech II. The soluble solids content (°Brix) was measured using a digital pocket refractometer (Atago U.S.A, Inc., Bellevue, WA) from 300 mL blueberry juice. Fruit weight (g) was measured using an analytical scale.

**3) Genotype-by-Environment (GxE) interactions**

As suggested in the ANOVA results, GxE in an important source of variation. In the Figure S3, we showed the phenotypic performance of 16 checks evaluated over the four regions in the Florida State. Importantly, we can observe crossover interactions, that ultimately indicates that the choice of the best genotype is determined by the environment.

Although GxE interaction in an important source of variation, it has not been formally modeled in the predictive models described. As described in the Table S1, we have unbalanced field experiments, making it challenging to properly model GxE effects. As described in our breeding pipeline, every year new genotypes are used to feed the calibration set. This is the current strategy used for the breeding program to update our model and keep it close to the breeding population (our ultimate target). During this process, advanced selections (promising genotypes) are extensively phenotyped for several traits and across multiple years and locations. On the one hand, this strategy connects the theory to the practice; but, on the other hand, it creates highly unbalanced datasets, which ultimately affects the convergence of more complex models. To circumvent issues at the inferential level caused by the omission of GxE interaction, we included eventual environmental sources of variations as cofactors in the model (eg.: age, location and year). We envision leveraging our phenotyping capacity by incorporating more common genotypes (statistical checks) across multiple years and locations. We hope that such approach can create all the necessary connections to properly model complex GxE interactions.

**
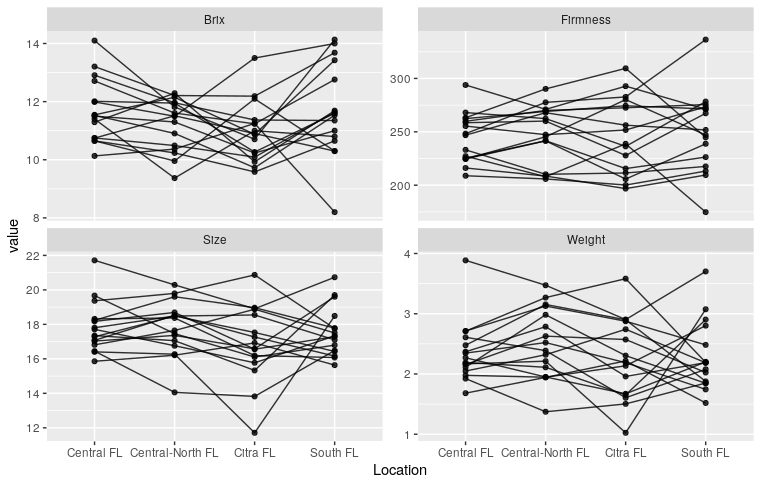
Figure S3.** Scatterplot of 16 statistical checks evaluated for four fruity-quality traits over four regions at the Florida State: Central FL, Citra, North FL and South FL.

**4) Predictive Ability**

Table S2. Predictive ability between two different genotype calling approaches (dosage and ratio) under two sequencing depth scenarios (6x and 60x) for four fruit quality traits in blueberry using 10-fold cross-validation. Results are means and, between brackets, the range observed.

| **Method** | **Depth** | **Firmness** | **Size** | **Weight** | **Brix** |
| --- | --- | --- | --- | --- | --- |
| Dosage | 6x | 0.44 [0.40-0.52] | 0.38 [0.29-0.49] | 0.47 [0.40-0.57] | 0.28 [0.18-0.47] |
| Dosage | 60x | 0.46 [0.41-0.55] | 0.40 [0.29-0.53] | 0.49 [0.40-0.56] | 0.29 [0.17-0.46] |
| Ratio | 6x | 0.45 [0.38-0.55] | 0.40 [0.31-0.48] | 0.48 [0.42-0.56] | 0.28 [0.18-0.43] |
| Ratio | 60x | 0.46 [0.40-0.54] | 0.39 [0.29-0.51] | 0.49 [0.41-0.56] | 0.29 [0.17-0.47] |

**Table S3.** Predictive ability reported in previous studies for fruit-quality traits, yield, and volatile organic compounds (VOCs) using tetraploid additive GBLUP. Previous studies considered only the calibration set in a 10-fold cross-validation scheme. Heritability (h^2^) are reported as genomic heritability accessed by using molecular markers scored in a tetraploid fashion.

| **Trait** | **Category** | **Seasons** | **Population Size** | **Predictive ability** | **h^2^** | **Reference** |
| --- | --- | --- | --- | --- | --- | --- |
| Soluble Solid | Fruit quality | 2014 | 1,847 | 0.28 | 0.215 | (de Bem Oliveira et al., 2019) |
| Size | Fruit quality | 2014,2015 | 1,847 | 0.40 | 0.216 | (de Bem Oliveira et al., 2019) |
| Firmness | Fruit quality | 2014,2015 | 1,847 | 0.46 | 0.351 | (de Bem Oliveira et al., 2019) |
| pH | Fruit quality | 2014 | 1,847 | 0.26 | 0.191 | (de Bem Oliveira et al., 2019) |
| Fruit Scar | Fruit quality | 2014,2015 | 1,847 | 0.47 | 0.494 | (de Bem Oliveira et al., 2019) |
| Fruit Weight | Fruit quality | 2014,2015 | 1,847 | 0.44 | 0.513 | (de Bem Oliveira et al., 2019) |
| Yield | Production | 2014,2015 | 1,847 | 0.35 | 0.374 | (de Bem Oliveira et al., 2019) |
| Flower Buds | Production | 2015 | 1,847 | 0.18 | 0.188 | (de Bem Oliveira et al., 2019) |
| (E)-2-Hexenal | VOCs | 2015 | 886 | 0.47 | 0.50 | (Ferrão et al., 2020) |
| 1-Hexanol | VOCs | 2015 | 886 | 0.46 | 0.49 | (Ferrão et al., 2020) |
| 2-Heptanone | VOCs | 2015 | 886 | 0.58 | 0.54 | (Ferrão et al., 2020) |
| 2-Nonanone | VOCs | 2015 | 886 | 0.67 | 0.77 | (Ferrão et al., 2020) |
| 2-Undecanone | VOCs | 2015 | 886 | 0.65 | 0.71 | (Ferrão et al., 2020) |
| D-limonene | VOCs | 2015 | 886 | 0.56 | 0.62 | (Ferrão et al., 2020) |
| Decanal | VOCs | 2015 | 886 | 0.56 | 0.62 | (Ferrão et al., 2020) |
| Eucalyptol | VOCs | 2015 | 886 | 0.49 | 0.50 | (Ferrão et al., 2020) |
| Geranyl acetone | VOCs | 2015 | 886 | 0.53 | 0.61 | (Ferrão et al., 2020) |
| Hexanal | VOCs | 2015 | 886 | 0.41 | 0.50 | (Ferrão et al., 2020) |
| Linalool | VOCs | 2015 | 886 | 0.53 | 0.58 | (Ferrão et al., 2020) |

5) Generalized Relationship Matrix (GRM) Dosage vs. Ratio

Table S4: Pearson’s correlation between off-diagonal values of genomic relationship matrices computed using dosage and ratio parameterizations under two sequencing depth scenarios (6x and 60x)

|  | **dosage_60x** | **ratio_60x** | **dosage_6x** | **ratio_6x** |
| --- | --- | --- | --- | --- |
| **dosage_60x** | 1.00 | 0.99 | 0.97 | 0.79 |
| **ratio_60x** |  | 1.00 | 0.96 | 0.79 |
| **dosage_6x** |  |  | 1.00 | 0.83 |
| **ratio_6x** |  |  |  | 1.00 |

**
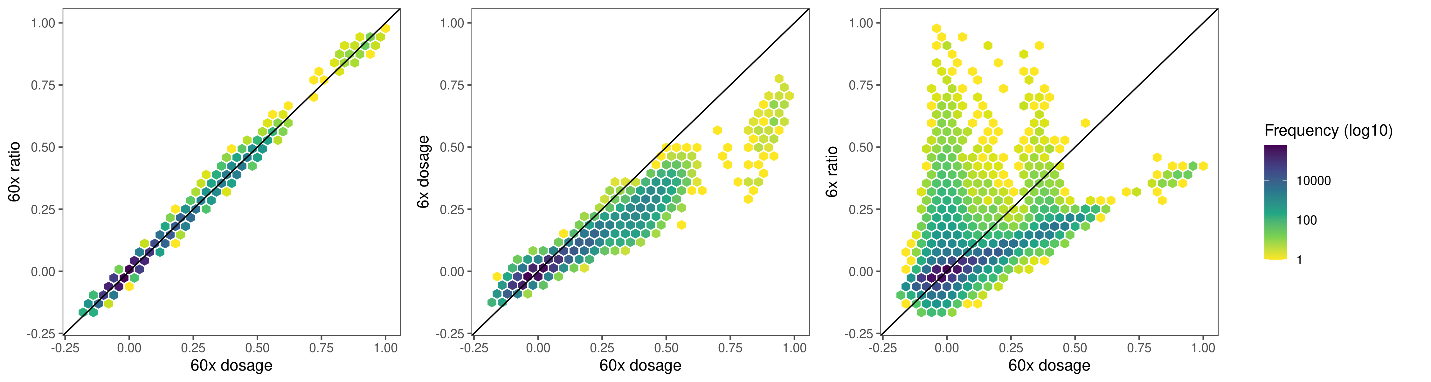
**

Figure S4. Scatterplot of off-diagonal values for the genomic relationship matrices computed using dosage and ratio parametrizations under two sequencing depth scenarios (6x and 60x). Diagonal lines represent the absolute similarity between scenarios.
